# Free‐breathing fat and R_2_* quantification in the liver using a stack‐of‐stars multi‐echo acquisition with respiratory‐resolved model‐based reconstruction

**DOI:** 10.1002/mrm.28280

**Published:** 2020-04-17

**Authors:** Manuel Schneider, Thomas Benkert, Eddy Solomon, Dominik Nickel, Matthias Fenchel, Berthold Kiefer, Andreas Maier, Hersh Chandarana, Kai Tobias Block

**Affiliations:** ^1^ Pattern Recognition Lab, Department of Computer Science Friedrich‐Alexander‐Universität Erlangen Nürnberg Erlangen Germany; ^2^ MR Applications Predevelopment Siemens Healthcare GmbH Erlangen Germany; ^3^ Center for Advanced Imaging Innovation and Research (CAI2R), Department of Radiology New York University School of Medicine New York NY USA; ^4^ MR R&D Collaborations Siemens Medical Solutions New York NY USA

**Keywords:** compressed sensing, free‐breathing fat/ $R_{2}^{*}$ quantification, multi‐echo 3D stack‐of‐stars GRE, nonalcoholic fatty liver disease, radial sampling, respiratory motion‐resolved reconstruction

## Abstract

**Purpose:**

To develop a free‐breathing hepatic fat and $R_{2}^{*}$ quantification method by extending a previously described stack‐of‐stars model‐based fat‐water separation technique with additional modeling of the transverse relaxation rate $R_{2}^{*}$.

**Methods:**

The proposed technique combines motion‐robust radial sampling using a stack‐of‐stars bipolar multi‐echo 3D GRE acquisition with iterative model‐based fat‐water separation. Parallel‐Imaging and Compressed‐Sensing principles are incorporated through modeling of the coil‐sensitivity profiles and enforcement of total‐variation (TV) sparsity on estimated water, fat, and $R_{2}^{*}$ parameter maps. Water and fat signals are used to estimate the confounder‐corrected proton‐density fat fraction (PDFF). Two strategies for handling respiratory motion are described: motion‐averaged and motion‐resolved reconstruction. Both techniques were evaluated in patients (*n* = 14) undergoing a hepatobiliary research protocol at 3T. PDFF and $R_{2}^{*}$ parameter maps were compared to a breath‐holding Cartesian reference approach.

**Results:**

Linear regression analyses demonstrated strong (*r* > 0.96) and significant (*P* ≪ .01) correlations between radial and Cartesian PDFF measurements for both the motion‐averaged reconstruction (slope: 0.90; intercept: 0.07%) and the motion‐resolved reconstruction (slope: 0.90; intercept: 0.11%). The motion‐averaged technique overestimated hepatic $R_{2}^{*}$ values (slope: 0.35; intercept: 30.2 1/s) compared to the Cartesian reference. However, performing a respiratory‐resolved reconstruction led to better $R_{2}^{*}$ value consistency (slope: 0.77; intercept: 7.5 1/s).

**Conclusions:**

The proposed techniques are promising alternatives to conventional Cartesian imaging for fat and $R_{2}^{*}$ quantification in patients with limited breath‐holding capabilities. For accurate $R_{2}^{*}$ estimation, respiratory‐resolved reconstruction should be used.

## 1 INTRODUCTION

Nonalcoholic fatty liver disease (NAFLD) refers to a form of chronic liver disease characterized by abnormal hepatic fat accumulation,^1^ which can progress to a spectrum of histologic features including steatosis, portal and lobular inflammation, ballooned hepatocytes, necrosis, and fibrosis.^2^ Liver biopsy allows for histologic assessment of liver injury and is the clinical standard for grading and staging liver diseases.^1^ However, it is an invasive procedure that suffers from sampling and interobserver variability, high costs, and postprocedural complications.^3^, ^4^

Non‐invasive assessment of hepatic fat accumulation can be achieved with MRI using chemical‐shift‐based Dixon techniques.^5^, ^6^, ^7^, ^8^, ^9^, ^10^, ^11^, ^12^, ^13^ These techniques encode water and fat in the time domain by acquiring the MR signal at different echo times (TE). By modeling additional confounding factors ( $T_{1}$, $T_{2}^{*}$, spectral complexity of fat, noise bias, and eddy currents), it becomes possible to use the estimated water and fat images for quantitative, un‐confounded proton‐density fat fraction (PDFF)^14^ estimation in liver tissue.^15^, ^16^, ^17^, ^18^, ^19^, ^20^, ^21^, ^22^, ^23^, ^24^ PDFF is a known biomarker for assessment of hepatic steatosis.^25^

Excessive iron deposition is another histological hallmark and associated with a higher risk of progression to advanced disease stages, such as cirrhosis or liver failure.^26^ Liver iron overload can also be detected using MRI because the hepatic iron content has been shown to linearly correlate with liver $R_{2}^{*}$ values at clinically relevant levels.^27^ Qualitatively, liver iron overload detection is possible using conventional $T_{2}^{*}$‐weighted imaging, or with in‐/opposed‐phase gradient‐echo (GRE) imaging.^26^ Quantitatively, iron deposition can be assessed using $R_{2}^{*}$ relaxometry techniques, which estimate the exponential decay rate of the GRE signal from multiple echoes.^28^, ^29^ Since $R_{2}^{*}$ is one of the confounders affecting fat fraction values (and vice versa), methods that simultaneously perform fat‐water separation and $R_{2}^{*}$ mapping for joint fat and iron quantification have been developed.^16^, ^18^, ^20^, ^21^

Conventionally, Cartesian sequences are used for PDFF or $R_{2}^{*}$ estimation.^15^, ^16^, ^17^, ^18^, ^19^, ^20^, ^21^, ^23^ However, Cartesian techniques can suffer from high motion sensitivity, which is why measurement times for abdominal imaging are typically restricted to 1 breath‐hold (BH). This poses a restriction on the achievable volumetric coverage, spatial resolution, or signal‐to‐noise ratio. Moreover, it restricts the method’s applicability in subjects who are unable to suspend respiration, such as sick, elderly, or pediatric patients. Radial sequences sample the k‐space center continuously, which makes them inherently more robust to motion than Cartesian sequences.^30^ This enables free‐breathing motion‐averaged^31^, ^32^ or motion‐resolved^33^ applications, which can be used to overcome the limitations associated with breath‐holding when quantifying PDFF^22^, ^34^ and $R_{2}^{*}$ ^35^, ^36^ in the abdomen.

The recently proposed Dixon‐RAdial Volumetric Encoding (Dixon‐RAVE) technique implements this strategy for free‐breathing fat‐water separation by combining radial sampling with iterative model‐based reconstruction, Compressed Sensing, and Parallel Imaging.^13^ In the current work, the Dixon‐RAVE method is extended by additionally modeling transverse $R_{2}^{*}$ relaxation, as required for accurate PDFF assessment. Hence, the proposed technique enables free‐breathing quantification of hepatic fat and $R_{2}^{*}$. Two strategies for handling respiratory motion are presented: motion‐averaged reconstruction and motion‐resolved eXtra‐Dimensional (XD) reconstruction. Both approaches are evaluated in vivo by comparing the estimated hepatic PDFF and $R_{2}^{*}$ values quantitatively with a breath‐holding Cartesian reference technique and a free‐breathing respiratory motion‐gated reconstruction at 3T.

## 2 THEORY

### 2.1 Inverse‐problem formulation and signal model

Spatially resolved estimates of the complex‐valued water (**W**) and fat content (**F**), and the real‐valued transverse signal relaxation rate ( $R_{2}^{*}$) can be calculated from the acquired complex radial k‐space data $Y_{c,t_{n}}$ by solving the following inverse reconstruction problem

(1) $${\text{argmin}}_{W,\;F,\;R_{2}^{*}}\sum_{c,t_{n}}\|E{\left(W,\;F,\;R_{2}^{*}\right)}_{c,t_{n}}-Y_{c,t_{n}}{\|}_{2}^{2}+\lambda_{W}\|S\left(W\right){\|}_{1}+\lambda_{F}\|S\left(F\right){\|}_{1}+\lambda_{R_{2}^{*}}{\left\|S\left(R_{2}^{*}\right)\right\|}_{1}.$$

Here, *E* corresponds to a signal model that is used to enforce data consistency for all receive coil elements *c* and echo times $t_{n}$, *n* = 1 to $N_{\mathrm{eco}}$. A single effective $R_{2}^{*}$ model is assumed. Compressed Sensing is incorporated through regularization terms that include sparsifying transforms *S*(.) of the water, fat, and $R_{2}^{*}$ maps. $\lambda_{W}$, $\lambda_{F}$ and $\lambda_{R_{2}^{*}}$ are the corresponding regularization weights. The signal model (or “forward operator") maps water, fat, and $R_{2}^{*}$ estimates to k‐space by applying coil sensitivity profiles $C_{c}$, a $B_{0}$ off‐resonance map (“field map") ***Φ***, and a multi‐peak fat model $D_{t_{n}}$ ^13^:

(2) $$E{\left(W,\;F,\;R_{2}^{*}\right)}_{c,t_{n}}=\mathrm{FT}\left(C_{c}e^{2\pi i\Phi t_{n}}e^{-R_{2}^{*}t_{n}}W\right)+D_{t_{n}}\mathrm{FT}\left(C_{c}e^{2\pi i\Phi t_{n}}e^{-R_{2}^{*}t_{n}}F\right).$$

The operator FT(.) performs a non‐uniform fast Fourier transform (NUFFT), which computes a conventional fast Fourier transformation (FFT) and a gridding procedure based on Kaiser‐Bessel interpolation kernel. The fat model accounts for the chemical shift of the fat peaks and uses the exact readout time of each k‐space sample, which results in inherent deconvolution of off‐resonant blurring of fat.^8^, ^13^ An analytical formulation of the gradient of the data‐consistency term within Equation (1) can be found online in the Supporting Information material.

Accurate quantitative fat fraction assessment requires careful consideration of several confounding factors.^14^ In the current approach, $T_{1}$ and noise biases are addressed using low flip angles and magnitude discrimination.^7^ $T_{2}^{*}$ decay and the multi‐peak fat spectrum are addressed directly in the signal model. Furthermore, calibration spokes are acquired to characterize and compensate for gradient delays and eddy‐current effects within the model‐based reconstruction.^37^ The confounder‐corrected PDFF is then calculated on a voxel‐by‐voxel basis using^7^, ^14^

(3) $${\mathrm{PDFF}}_{i}=\left\{\begin{array}{cc} \frac{\left|F_{i}\right|}{\left|F_{i}+W_{i}\right|}, & \text{if}\;|F_{i}\left|\geq \right|W_{i}|\\ 1-\frac{\left|W_{i}\right|}{\left|F_{i}+W_{i}\right|}, & \text{otherwise} \end{array}\right.$$

where *i* = 1, …, *N* and *N* is the number of voxels. The $R_{2}^{*}$ map can be used to estimate tissue iron content because the confounders multi‐peak fat spectrum and $B_{0}$ variations are addressed and because the complex‐fitting technique is not affected by noise floor effects.^26^ In the following, 2 variants of the proposed free‐breathing fat and $R_{2}^{*}$ quantification technique are discussed.

### 2.2 Motion‐averaged reconstruction

It is assumed that radial stack‐of‐stars data $Y_{c,t_{n}}$ is acquired during free‐breathing with $N_{\mathrm{read}}$ readout samples, $N_{\mathrm{proj}}$ radial views, $N_{z}$ partitions, $N_{\mathrm{coil}}$ channels, and $N_{\mathrm{eco}}$ echoes. The proposed technique uses this data of size $\left[N_{\mathrm{read}},\;N_{\mathrm{proj}},\;N_{z},\;N_{\mathrm{coil}},\;N_{\mathrm{eco}}\right]$ and estimates PDFF and $R_{2}^{*}$ maps of size $\left[N_{x},\;N_{y},\;N_{z}\right]$ by solving Equation (1) and (3). Here, $N_{x}$ and $N_{y}$ are the image dimensions, and $N_{z}$ is the number of slices. Spatial total variation (TV) (spatial finite differences) is used as sparsifying transform *S*(.). Because the estimated parameter maps are reconstructed from all k‐space data (depending on a range of different respiratory states during the free‐breathing acquisition), this approach is referred to as “motion‐averaged" reconstruction in the following.

### 2.3 Motion‐resolved XD reconstruction

The radial k‐space sampling scheme utilized in the technique is inherently robust to motion. However, deep breathing can lead to residual artifacts in the form of blurring or streaking.^13^ More importantly, for $R_{2}^{*}$ mapping motion sensitivity is additionally problematic due to inconsistencies in the magnitude and phase information at each spatial location during the free‐breathing scan.^35^ Therefore, the reconstruction approach can be extended to resolve an additional motion dimension. Similar to the previously proposed XD‐GRASP method for fat‐suppressed free‐breathing imaging^33^ and the original XD‐Dixon‐RAVE method for qualitative fat‐water separation,^13^ the continuously acquired data can be sorted into $N_{\mathrm{bin}}$ respiratory states. To this end, a variable‐amplitude binning strategy, which ensures that the number of spokes (ie, the scan time) is uniformly distributed over all states (uniform binning), is applied. The resulting undersampled multi‐dimensional data of size $\left[N_{\mathrm{read}},\;N_{\mathrm{proj}}/N_{\mathrm{bin}},\;N_{z},\;N_{\mathrm{coil}},\;N_{\mathrm{eco}},\;N_{\mathrm{bin}}\right]$ can then be reconstructed, yielding parameter maps of size $\left[N_{x},\;N_{y},\;N_{z},\;N_{\mathrm{bin}}\right]$. For this technique, temporal TV along the respiratory motion bin dimension is used as sparsifying transform *S*(.) in Equation (1) (instead of the spatial TV regularization that is used for the motion‐averaged reconstruction). The required respiratory self‐navigation signal is extracted from central k‐space points by estimating interpolated and normalized projection profiles along the *z* axis, followed by principal component analysis (PCA).^33^, ^38^ Signals from principal components containing the dominant part of motion are then automatically selected using coil clustering,^39^ averaged, and used as surrogate for the respiratory state. The parameter maps corresponding to the frame containing the least amount of motion are selected and used for qualitative and quantitative evaluation. The frame with best motion consistency is found by considering the amplitudes of the respiration signal samples corresponding to a respective frame, and by then estimating the variance of that values. The frame with the smallest variance in the respiration signal is selected.

### 2.4 Sequence description

The proposed technique utilizes a custom‐developed bipolar T1‐weighted stack‐of‐stars 3D GRE sequence that acquires 6 echoes within 1 repetition time (TR).^13^, ^30^ Radially sampled data are acquired in the $k_{x}$‐ $k_{y}$‐plane using Golden‐Angle ordering.^40^ For every projection angle, all linearly ordered partitions ( $k_{z}$ direction) are acquired sequentially before rotating to the next radial view. A new sample of the extracted respiratory self‐gating signal is calculated every time the k‐space center is sampled. Therefore, the described re‐ordering scheme allows retrospective binning of all acquisitions for a specific radial view into the same bin, that is, all partitions of 1 specific radial angle are sorted into the same respiration frame. Furthermore, the sequence applies small blip gradients in $k_{x}$ and $k_{y}$ direction between the readouts of 1 echo train in order to slightly rotate subsequent echoes. This strategy increases incoherence along the echo dimension, which leads to reduced streaking artifacts.^13^ k‐Space shifts in the measurement data due to gradient imperfections and eddy currents are corrected by repeatedly acquiring calibration lines in opposing direction along the $k_{x}$‐ and $k_{y}$‐axis^22^, ^24^, ^37^ for all echoes prior to the actual data acquisition. In total, 10 calibration lines are acquired for each direction (positive and negative $k_{x}$‐ and $k_{y}$‐axis). Expected k‐space shifts can be estimated by cross‐correlating calibration scans with opposing polarity. k‐Space shift values are estimated separately for each echo, but the same shifts are applied to all receiver channels. Signal‐delay compensation is then incorporated into the model‐based reconstruction by considering the k‐space shifts in the gridding procedure of the NUFFT within Equation (2).

## 3 METHODS

### 3.1 Implementation details

Spatially resolved $B_{0}$ inhomogeneity information is crucial for robust water and fat separation. Therefore, images are calculated for every acquired echo using conventional NUFFT before evaluating Equation (1). These images are then used for estimation of a field map ***Φ*** based on a regularized field‐map formulation,^41^ which is initialized using a discretized graph‐cut solution.^10^ This approach additionally yields water, fat, and $R_{2}^{*}$ maps, which can be used to initialize the proposed iterative reconstruction. Moreover, coil sensitivity maps $C_{c}$, which are incorporated in the forward operator $E{\left(W,\;F,\;R_{2}^{*}\right)}_{c,t_{n}}$ (Equation (2)), are estimated from the individual regridded coil images using the adaptive array combining approach by Walsh et al.^42^ The k‐space fat model formulation $D_{t_{n}}$ is based on a 6‐peak fat model.^13^, ^43^, ^44^

Because the implemented iterative reconstruction scheme is computationally demanding, various measures have been taken to reduce processing times. Coil‐array compression using PCA^45^ is applied partition‐wise on the acquired k‐space data to compress the multi‐channel datasets into the first 8 (for the motion‐averaged reconstruction) or 4 (for the motion‐resolved reconstruction) eigenmodes. The number of eigenmodes was kept fixed for all subjects of this study. Moreover, an inverse FFT is performed along the partition dimension before applying the proposed technique to enable parallelized reconstruction of individual slices. For the used protocol and reconstruction parameters, the cost function is typically more sensitive to changes in the water or fat signal than to changes in $R_{2}^{*}$. Therefore, a data‐driven $R_{2}^{*}$ scaling factor has been introduced to ensure that the L2‐norms of the partial derivatives with respect to water, fat, and $R_{2}^{*}$ are in a similar range. This balances the influence of the individual parameters on the cost function and reduces the required number of iterations. The scaling factor is calculated as follows. First, the partial derivatives of the data‐fidelity term of Equation (1) with respect to water‐fat and $R_{2}^{*}$ based on the initial water, fat and $R_{2}^{*}$ values are calculated. The scaling factor can then be calculated as the ratio of the ℓ2‐norms of the partial derivatives of water or fat and $R_{2}^{*}$.

The proposed reconstruction has been implemented in Matlab (The MathWorks, Inc., Natick, Massachusetts) with use of an L‐BFGS (Limited‐memory Broyden‐Fletcher‐Goldfarb‐Shanno) minimizer.^46^

### 3.2 In vivo study

The performance of the technique was assessed qualitatively and quantitatively in vivo in *n* = 14 patients (7 male, 7 female, age: 57 ± 18 years, weight: 77.4 ± 17.5 kg, BMI: $27.5\;\pm \;5.7\;\text{kg}\;m^{-2}$). All patients underwent clinical MR elastography and were enrolled in an IRB‐approved and HIPAA‐compliant hepatobiliary research protocol. Based on measurements in the right liver lobe (avoiding visible blood vessels) using conventional Cartesian multi‐echo MRI during breath‐holding, 7 patients had hepatic fat overload, and 2 patients had liver iron overload. Here, cutoff values proposed by Zhan et al^47^ (PDFF: 3.4%; $R_{2}^{*}$: 60.5 1/s) were applied. Data were acquired pre‐contrast on a 3T scanner (MAGNETOM Skyra, Siemens Healthcare, Erlangen, Germany) using a spine and body array coil. The described stack‐of‐stars 3D GRE sequence was used to acquire one 6‐echo dataset per patient with ${\mathrm{TE}}_{1}\;=\;1.23\;\text{ms}$ and echo spacing ΔTE = 1.23 ms. Other imaging parameters included TR = 8.76 ms, $\text{flip angle}\;=\;5^{\circ }$, field‐of‐view = 410 × 410 × 260 mm, base resolution = 256 × 256, slice thickness = 5 mm, and readout bandwidth = 1090 Hz/px. The number of slices ranged from 48 to 64 to achieve full liver coverage. Corresponding scan times ranged from 3 minutes 17 seconds to 4 minutes 13 seconds. Four hundred radial views (projections) were measured. Subsequent echoes were rotated by $1.5^{\circ }$. Every acquired radial dataset was reconstructed as follows:

1. Using the respiratory motion‐averaged technique, applied to all 400 (fully sampled) acquired projections (termed “Motion‐averaged"). The regularization weights were set to zero for this reconstruction ( $\lambda_{W}\;=\;\lambda_{F}\;=\;\lambda_{R_{2}^{*}}\;=\;0$).
2. Using the proposed respiratory motion‐resolved reconstruction, applied to all 400 acquired projections (fully sampled) and using $N_{\mathrm{bin}}\;=\;4$ frames (termed “Motion‐resolved XD"). Regularization weights were chosen heuristically.

The “Motion‐resolved XD" approach is particularly computationally demanding. To illustrate the advantages of the method compared to a computationally less complex strategy, acquired radial datasets from all 14 patients were additionally reconstructed by means of a method that applied retrospective hard‐gating. Therefore, k‐space data of the frame (out of $N_{\mathrm{bin}}\;=\;4$ frames) containing the least amount of motion (ie, an acceptance rate of 25%) was reconstructed using conventional NUFFT, followed by state‐of‐the‐art image‐based fat‐water separation.^10^, ^41^ The remaining 3 bins (75% of the acquired spokes) were neglected, and in the following this method is referred to as “Motion‐gated NUFFT (25% accept.)."

To give further insight into differences of motion‐resolved versus motion‐gated, as well as NUFFT reconstruction and image‐based water‐fat separation versus model‐based reconstruction techniques, 1 representative patient was reconstructed using 2 further motion‐gated reconstructions. The first method reconstructed the same frame using model‐based iterative fat‐water separation (termed “Motion‐gated Model‐based (25% accept.)"). This was achieved by using the motion‐resolved reconstruction framework without any regularization ( $\lambda_{W}\;=\;\lambda_{F}\;=\;\lambda_{R_{2}^{*}}\;=\;0$). The second method split up the acquired data into $N_{\mathrm{bin}}\;=\;2$ frames, and reconstructed the frame with best motion consistency using NUFFT and conventional fat‐water separation.^10^, ^41^ This method is referred to as “Motion‐gated NUFFT (50% accept.)." For all motion‐gated reconstructions, the frame with best motion consistency was found using the same technique and the same self‐navigation signal as in the proposed motion‐resolved XD reconstruction. The same field map estimation technique^10^ and fat model^44^ were used for all described reconstructions.

The radial datasets were reconstructed on a server with 2 Intel Xeon Gold 6238 CPUs (2.10 GHz and 22 cores each). Measured reconstruction times for an example case were 46 minutes, 1 hour 35, and 25 minutes for “Motion‐averaged," “Motion‐resolved XD," and “Motion‐gated NUFFT (25% accept.)."

The proposed techniques were compared to a clinically validated^21^, ^48^ breath‐holding Cartesian reference method, which used a conventional 3D GRE sequence Volumetric Interpolated Breath hold Examination (VIBE) along with a multi‐step adaptive PDFF and $R_{2}^{*}$ fitting technique,^20^ applying a time‐domain calibration of the fat signal dephasing that was optimized for liver applications.^49^ Protocol parameters of the Cartesian acquisition were kept similar to the radial protocol where technically possible, but the acquisition time was reduced to about 12 seconds and data were acquired during breath‐holds. The Cartesian data acquisition was accelerated 4‐fold using CAIPIRINHA^50^ (2‐fold acceleration along $k_{y}$ as well as $k_{z}$). In the following, the reference method is referred to with “BH Cartesian." Table 1 summarizes the protocol parameters for both radial and Cartesian acquisitions.

**Table 1:** Protocol parameters for (left) the free‐breathing radial Dixon‐RAVE and (right) the breath‐hold Cartesian reference acquisition

Parameters	Field strength: 3 T	
	Protocol 1: Radial	Protocol 2: Cartesian
Sequence	Dixon‐RAVE	VIBE
Number of echoes	6	6
First echo ${\text{TE}}_{1}$ (ms)	1.23	1.09
Echo spacing ΔTE (ms)	1.23	1.23
Repetition time TR (ms)	8.75	9
Flip angle	$5^{\circ }$	$4^{\circ }$
Field‐of‐view ( ${\text{mm}}^{3}$)	410 × 410 × 260	332 × 380 × 192
Base resolution (pixels)	256 × 256	160 × 140
Pixel size ( ${\text{mm}}^{2}$)	1.6 × 1.6	1.2 × 1.2 (interp.)^a^
Slice thickness (mm)	5	6 (acquired); 3 (interp.)^c^
Slice resolution (%)	100	50
Readout mode	bipolar	bipolar
Bandwidth (Hz/px)	1090	1080
Number of slices	48‐64	64‐96
Averages	1	1
Radial views	400	‐
Radial sampling	Golden‐angle	‐
Total measurement time	3 minutes 17 second‐4 minutes 13 seconds	14 seconds‐20 seconds^b^

*Note*: For the radial acquisition, the total measurement time includes the gradient calibration time.

a Acquired pixel size: 2.4 × 2.4 mm.

b 4‐fold accelerated using CAIPIRINHA (twofold acceleration along $k_{y}$ as well as $k_{z}$).

c The Cartesian protocol applied a “slice resolution" of 50%, ie, only 50% of the phase encoding steps are acquired and zero‐padded, resulting in 2 times higher nominal slice resolution (interpolated).

### 3.3 Selection of regularization weights

Since “Motion‐averaged" was applied to fully sampled data, no spatial TV regularization was applied for this approach ( $\lambda_{W}\;=\;\lambda_{F}\;=\;\lambda_{R_{2}^{*}}\;=\;0$). Heuristic observations showed that, for the motion‐resolved XD reconstructions, temporal regularization weights for water and fat ( $\lambda_{W}$ and $\lambda_{F}$) and the regularization weight for $R_{2}^{*}$ ( $\lambda_{R_{2}^{*}}$) can be optimized rather independently of each other. Therefore, the following strategy for finding appropriate regularization weights has been pursued. First, “Motion‐resolved XD" was applied to an example case with varying $\lambda_{W}$ and $\lambda_{F}$ while $\lambda_{R_{2}^{*}}$ was set to zero (see Supporting Information Figure S1). Weights for water and fat were kept equal for all reconstructions ( $\lambda_{W}\;=\;\lambda_{F}$), and regularization weights of $\lambda_{W}\;=\;\lambda_{F}\;=\;0.4$ were selected based on visual inspection. Then, the technique was applied using the chosen regularization weights for water and fat and with varying $\lambda_{R_{2}^{*}}$ (see Supporting Information Figure S2). The weight $\lambda_{R_{2}^{*}}\;=\;0.01$ was chosen empirically. Values for $\lambda_{W}$, $\lambda_{F}$, and $\lambda_{R_{2}^{*}}$ in a range from 0 to 0.8 were considered during this selection process. The regularization factors $\lambda_{W}$/ $\lambda_{F}$ were selected by 2 MR physicists with 6 and 7 years of professional experience and a board‐certified radiologist with 12 years of experience by visual inspection of the PDFF parameter maps, respectively. The used assessment criteria were streaking artifact suppression, homogeneity of the respective parameter maps, and temporal fidelity across individual frames. The regularization weight $\lambda_{R_{2}^{*}}$ was chosen by inspecting $R_{2}^{*}$ using the same approach. The generalizability of the estimated values to different datasets was verified by running reconstructions of other datasets of the study subjects with different PDFF/ $R_{2}^{*}$ values for a range of regularization parameters.

### 3.4 Quantitative evaluation

For every patient and reconstruction method, circular volume of interest (VOI) (ranging from 138 to 210 voxels) were drawn in the Couinaud liver segments II, III, V, VI, VII, and VIII (avoiding visible blood vessels). One VOI that comprises 2 consecutive slices was drawn per segment and then mean PDFF and $R_{2}^{*}$ values in the VOIs were calculated. VOI evaluation was skipped for the $R_{2}^{*}$ parameter maps for Couinaud segments VII and VIII because in the upper parts of these 2 segments, the estimated $R_{2}^{*}$ maps from the radial acquisitions suffer from strongly increased $R_{2}^{*}$ values in some of the patients.

Linear‐regression and Bland‐Altman analyses were performed to compare “Motion‐averaged" with “BH Cartesian," “Motion‐gated NUFFT (25% accept.)" with “BH Cartesian," and “Motion‐resolved XD" with “BH Cartesian." For all 3 comparisons, slope and intercept of the regression lines (including 95% confidence intervals) as well as Pearson’s linear correlation coefficients *r* (including 95% confidence intervals) were calculated. The mean absolute errors (MAEs) between the respective techniques were determined. Additionally, *P* values describing the probability that there is no linear correlation between the methods (null‐hypothesis) were given (*P* < .01 was considered statistically significant). Moreover, for all comparisons, biases as well as 95% limits of agreement (LoA) were estimated (including 95% confidence intervals). All statistical calculations were performed using the Matlab Statistics and Machine Learning Toolbox (The MathWorks, Inc., Natick, Massachusetts).

## 4 RESULTS

**Figure 1:**
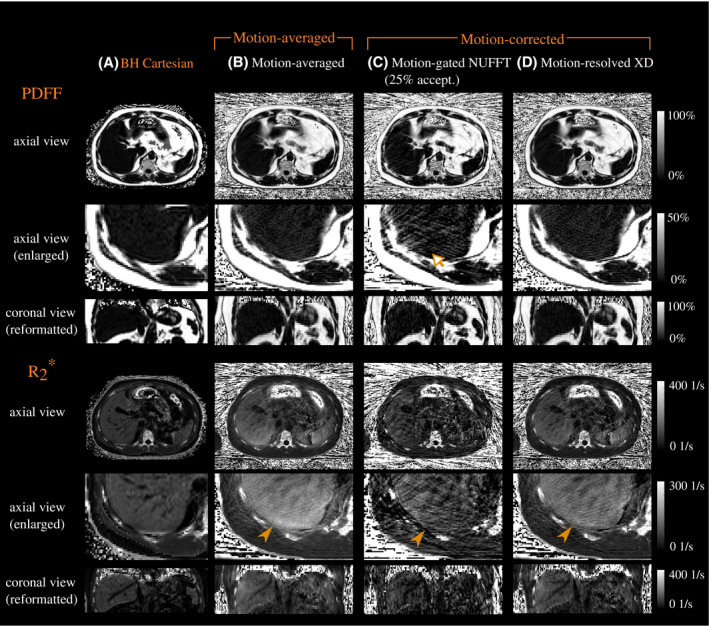
(Top) PDFF (with magnification) and (bottom) $R_{2}^{*}$ maps for (A) the breath‐held Cartesian reference technique, (B) the motion‐averaged reconstruction, (C) motion‐gating followed by conventional NUFFT and image‐based water‐fat separation, and (D) motion‐resolved XD reconstruction. The motion‐corrected parameter maps depict frame 2 (end‐expiration is frame 1). Visible streaking artifacts were observed in the PDFF maps from “Motion‐gated NUFFT (25% accept.)" (see arrow). Values in $R_{2}^{*}$ maps from “Motion‐averaged" were elevated compared to $R_{2}^{*}$ map values from “BH Cartesian," especially in the posterior segments. Opposed to that, $R_{2}^{*}$ map values of “Motion‐resolved XD" were visually more consistent with respect to the Cartesian reference (see filled arrow heads)

Figure 1 shows in vivo parameter maps from a patient with slightly elevated iron content in the liver. Comparable visual quality of the PDFF map in hepatic tissue was obtained with the techniques “BH Cartesian," “Motion‐averaged," and “Motion‐resolved XD." The “Motion‐gated NUFFT (25% accept.)" technique resulted in visible streaking artifacts in the PDFF maps in hepatic tissue (see arrow). $R_{2}^{*}$ map values of “Motion‐averaged" were elevated compared to $R_{2}^{*}$ map values from “BH Cartesian." In contrast, transverse relaxation map values of “Motion‐resolved XD" were visually more consistent with those from “BH Cartesian," especially in the posterior segments (see arrows). $R_{2}^{*}$ map values of “Motion‐gated NUFFT (25% accept.)" suffered from streaking. The respiratory position “end‐expiration" (bin 1 or 2) resulted in the most favorable image quality and quantitative mapping performance.

Supporting Information Figure S3 includes an animation showing all slices of example parameter maps from “Motion‐resolved XD" and “Motion‐averaged." Supporting Information Figure S4 includes a similar animation from an example patient with elevated PDFF as well as $R_{2}^{*}$.

**Figure 2:**
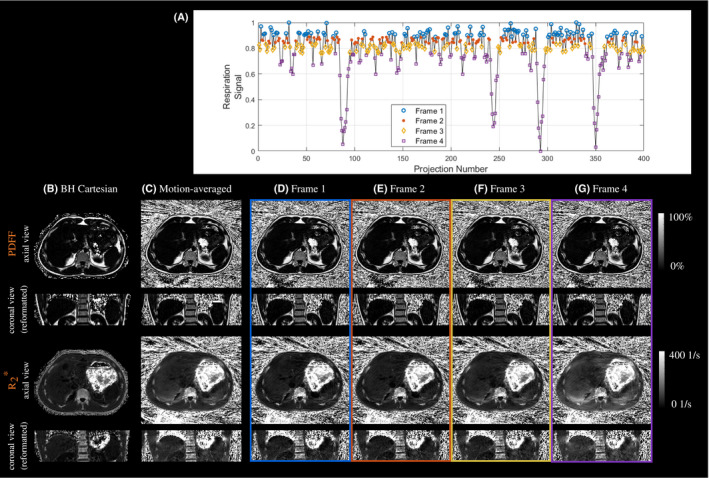
Estimated respiratory signal (A), and PDFF (top) and $R_{2}^{*}$ (bottom) maps for (B) the breath‐held Cartesian reference, and (C) the free‐breathing stack‐of‐stars data reconstructed using the motion‐averaged reconstruction. Motion‐resolved XD reconstruction for frame 1 (end‐expiration) to frame 4 (end‐inspiration) are shown in (D)‐(G). The motion‐resolved XD parameter maps corresponding to frame 2 are selected for quantitative evaluation

Figure 2 gives further insight into the motion sensitivity of estimated PDFF and $R_{2}^{*}$ maps by comparing a motion‐averaged reconstruction with the individual motion‐resolved frames estimated using “Motion‐resolved XD" for 1 exemplary subject. Hepatic $R_{2}^{*}$ values in frames reconstructed from data with best respiratory motion consistency (here: frame 1 and 2; frame 1 corresponds to end‐expiration) were lower compared to the motion‐averaged parameter maps. In contrast, hepatic $R_{2}^{*}$ values in the frame containing most of the motion (here: frame 4, which corresponds to end‐inspiration) were elevated compared to the motion‐averaged reconstruction. Comparable PDFF map quality was observed for the motion‐averaged and all frames of the motion‐resolved techniques. Supporting Information Figure S5 depicts the same comparison for a patient with slightly elevated $R_{2}^{*}$ values.

**Figure 3:**
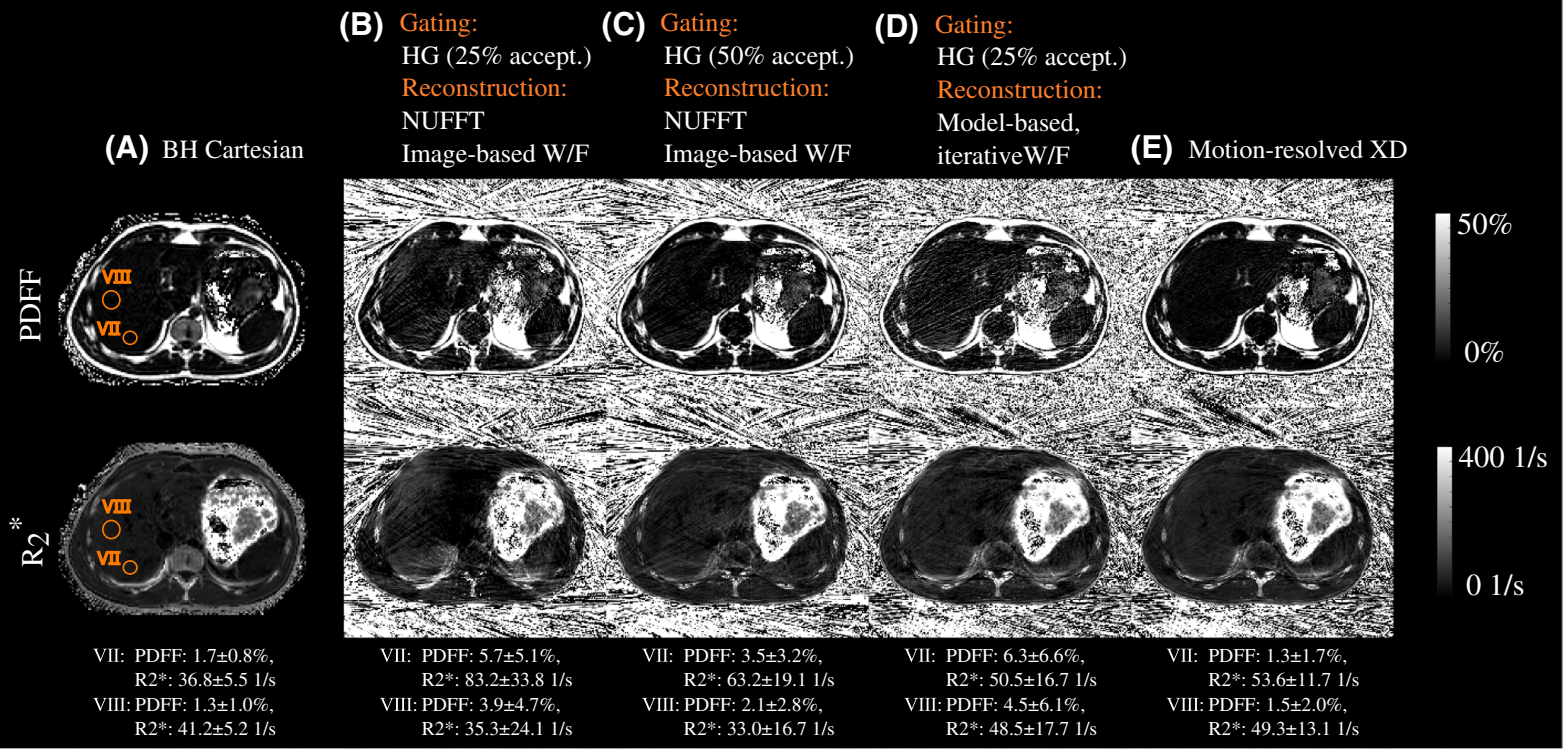
PDFF (top) and $R_{2}^{*}$ (bottom) maps of a 25‐year old patient (male, BMI: $24.6\;\text{kg}\;m^{-2}$) from (A) the breath‐held Cartesianeference scan, and (B)‐(E) the free‐breathing stack‐of‐stars acquisition (end‐expiratory frame). The radial dataset was reconstructed (B) using motion‐gating (25% acceptance rate) followed by NUFFT and image‐based water‐fat separation, (C) using motion‐gating (50% acceptance rate) followed by NUFFT and image‐based water‐fat separation, (D) using motion‐gating (25% acceptance rate) followed by model‐based water‐fat separation ("Motion‐resolved XD" with $\lambda_{W}\;=\;\lambda_{F}\;=\;\lambda_{R_{2}^{*}}\;=\;0$), and (D) using motion‐resolved XD reconstruction. Two exemplary VOIs were drawn in the parameter maps of “BH Cartesian" in the Couinaud liver segments VII and VIII. HG, hard‐gating

Figure 3 illustrates the advantages of the proposed “Motion‐resolved XD" reconstruction in comparison to 3 computationally less expensive motion‐resolved, free‐breathing quantification techniques. Performing motion‐gating along with NUFFT and image‐based fat‐water separation using an acceptance rate of 25% led to inhomogeneous maps containing strong streaking artifacts, especially for $R_{2}^{*}$. Using the same reconstruction technique with an acceptance rate of 50% led to a visible reduction of the streaking. The method combining motion‐gating with model‐based iterative fat‐water separation yielded PDFF maps with increased streaking artifact level and less homogeneous $R_{2}^{*}$ maps in the liver. The proposed “Motion‐resolved XD" reconstruction created PDFF maps with reduced streaking artifact level and more homogeneous $R_{2}^{*}$ maps, yielding parameter maps that were most consistent with maps from “BH Cartesian."

**Figure 4:**
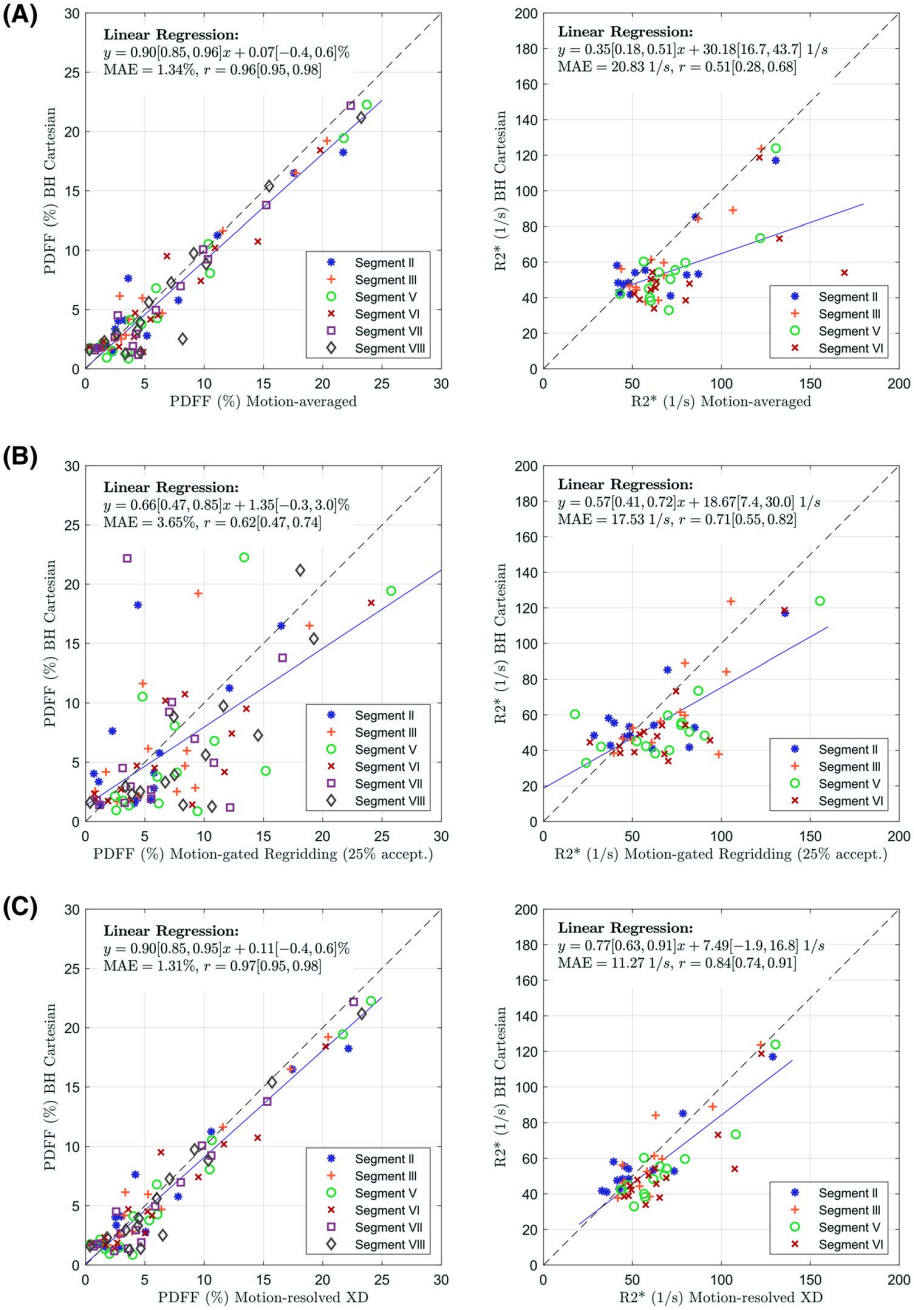
Correlation plots for the measured (left) PDFF and (right) $R_{2}^{*}$ values comparing (A) motion‐averaged reconstructions to the Cartesian reference, (B) motion‐gated (25% acceptance rate) reconstructions followed by NUFFT and image‐based water‐fat separation to the Cartesian reference, and (C) motion‐resolved XD reconstructions to the Cartesian reference

Linear regression analysis (see Figure 4) as well as Bland‐Altman plots (see Supporting Information Figure S6) of the performed patient study shown in Figure 4 confirmed the qualitative visual observations. For PDFF, strong (*r* > 0.96) and significant (*P* ≪ .01) correlations were observed between “Motion‐averaged" and "BH Cartesian" (slope: 0.9; intercept: 0.1%, Figure 4A), as well as “Motion‐resolved XD" and “BH Cartesian" (slope: 0.9; intercept: 0.1%, Figure 4C). Slightly more variation was observed between “Motion‐gated NUFFT (25% accept.)" and “BH Cartesian" (slope: 0.7; intercept: 1.4%, *r* > 0.62, *P* ≪ 0.01, Figure 4B). Similar biases in PDFF values were observed for all 3 reconstruction techniques compared to “BH Cartesian" (0.6%, 1.1% and 0.6% for “Motion‐averaged," “Motion‐gated NUFFT (25% accept.)," and “Motion‐resolved XD"). In contrast, “Motion‐averaged" strongly overestimated $R_{2}^{*}$ values compared to “BH Cartesian," especially in segments V and VI (slope: 0.35; intercept: 30.2 1/s; *r*: 0.51; *P* ≪ .01, Figure 4A; bias: 32.3 1/s, Supporting Information Figure S6A). Motion compensation using “Motion‐gated NUFFT (25% accept.)" (slope: 0.57; intercept: 18.7 1/s; *r*: 0.71; *P* ≪ .01, Figure 4B; bias: 14.4 1/s, Supporting Information Figure S6B) and using “Motion‐resolved XD" (slope: 0.77; intercept: 7.5 1/s; *r*: 0.84; *P* ≪ .01, Figure 4C; bias: 16.9 1/s, Supporting Information Figure S6C) improved the $R_{2}^{*}$ value consistency with “BH Cartesian."

## 5 DISCUSSION

This work describes a novel approach for free‐breathing hepatic fat and $R_{2}^{*}$ quantification using a motion‐robust radial multi‐echo acquisition and model‐based reconstruction that accounts for off‐resonant spectral components of fat. Good agreement of the motion‐averaged and motion‐resolved free‐breathing PDFF quantification relative to the Cartesian reference technique was observed. Therefore, if only PDFF quantification is of interest, both reconstruction techniques (motion‐averaged and motion‐resolved) can be used. In contrast, the motion‐averaged reconstruction approach failed to estimate quantitative $R_{2}^{*}$ values accurately, indicating higher motion sensitivity of this parameter. Therefore, we suggest that the respiration‐resolved reconstruction variant should be used if both PDFF and $R_{2}^{*}$ parameter maps are required.

Remaining differences between PDFF values from the proposed approach and the Cartesian reference technique might result from the differences in the applied multi‐peak fat models, or might be caused by streaking artifacts related to the radial acquisition scheme of the k‐space data. Residual streak artifacts are a common problem with radial acquisition schemes, especially in abdominal applications where the arms of the patients can enter areas of the magnetic field with strongly compromised $B_{0}$ homogeneity. This can result in visible streak artifacts that radiate from the arms and contaminate the region‐of‐interest. Hence, the location and strength of the streaks can differ from patient to patient, depending on the patient size, height, and placement. Since reliable NAFLD assessment requires high measurement accuracy for small fat fraction values, future work should focus on improving the PDFF value accuracy for small PDFF values, for example, by exploring various strategies for reducing streaking artifacts. For example, streaking suppression can be achieved by automatically excluding certain coils containing high levels of streaking during reconstruction.^51^, ^52^, ^53^

The linear regression analysis for $R_{2}^{*}$ between “BH Cartesian" and “Motion‐resolved XD" estimated a slope of 0.77, indicating residual differences between the 2 methods. These remaining differences between the motion‐resolved $R_{2}^{*}$ maps and the Cartesian reference could result from intra‐bin motion artifacts or from imperfections of the respiratory signals. Inspection of the reconstructed respiratory‐resolved images revealed that accurate extraction of the respiratory surrogate signal is challenging in some cases (especially in obese patients). More advanced algorithms for extraction or filtering of the self‐navigation signal (eg, Refs. 54, 55) or use of external respiration sensors (eg, Ref. 56) could help to further improve the overall quantitative $R_{2}^{*}$ mapping performance. Since the performance of the motion‐resolved reconstruction depends on the quality of the extracted respiratory signal, closer investigation of different breathing patterns should be subject of future work. Additional factors leading to differences in $R_{2}^{*}$ between “BH Cartesian" and “Motion‐resolved XD" include the differences in the applied multi‐peak fat model between the proposed and the reference technique, or VOI placement discrepancies. Moreover, residual streaking artifacts visible even in the motion‐resolved $R_{2}^{*}$ maps may increase the quantitative parameter map uncertainty. Furthermore, the radial and Cartesian acquisitions used slightly different in‐plane resolution. However, we do not think that this minor difference in acquisition parameters leads to a systematic bias in quantitative $R_{2}^{*}$ map values, but expect that the $R_{2}^{*}$ accuracy is much more affected by residual streak artifacts.

Less correlation and larger LoA in $R_{2}^{*}$ parameter map values at a group level were observed when comparing “Motion‐gated NUFFT (25% accept.)" to “BH Cartesian" as opposed to comparing “Motion‐resolved XD" to “BH Cartesian" (see Figure 4B,C and Supporting Information Figure S6B,C). This is mainly due to an increased residual streaking artifact level due to undersampling in the motion‐gated $R_{2}^{*}$ maps. Accepting only 25% of the data (100 out of 400 radial views) corresponds to an undersampling factor of approximately (256*π*/2)/100≈4 in this case. One possibility to reduce the streaking in the motion‐gated $R_{2}^{*}$ maps would be to acquire more data, thus longer acquisition times would have to be applied. Another option would be to increase the acceptance rate (see results for “Motion‐gated NUFFT (50% accept.)"). For example, Zhong et al^35^ reported good agreement in a patient study between a Cartesian reference approach and a motion‐gated free‐breathing stack‐of‐radial PDFF and $R_{2}^{*}$ estimation technique using a self‐gating acceptance rate of 40%. However, in the case of deep breathing, this would potentially lead to increased intra‐bin motion artifacts. In the performed in vivo study, segments VII and VIII were omitted from the quantitative evaluation of $R_{2}^{*}$ due to residual artifacts in the $R_{2}^{*}$ maps in the upper parts of these segments. Future work should focus on improving $R_{2}^{*}$ map quality from free‐breathing data in these areas.

Comparing the linear regression analyses of the proposed motion‐averaged and motion‐resolved reconstructions of the radial data with “BH Cartesian" revealed that respiratory motion causes a substantial bias in estimated $R_{2}^{*}$ values. If not corrected for, respiratory motion leads to data inconsistencies in magnitude and phase due to the varying motion states that are mixed. These inconsistencies might, for example, be caused by $B_{0}$ field variations due to respiration‐induced susceptibility changes. As a result, the MR signal decays faster. Fitting the signal model to the echo data then leads to artificially elevated $R_{2}^{*}$ estimation. Moreover, the motion might lead to "smearing" along the direction of motion, resulting in mixture of the values. Additionally, residual streaking or blurring artifacts due to deep breathing might lead to further uncertainty in the estimated transverse relaxation values from the motion‐averaged reconstruction. Due to the form of the signal model and cost function, the water and fat parameters are less susceptible to signal cancellation due to inconsistencies in magnitude and phase, and hence PDFF is less susceptible to respiratory motion. These results are in agreement with results from the study of Zhong et al,^35^ which—to the best of our knowledge—is the only previous study that simultaneously estimates PDFF and $R_{2}^{*}$ from radial data. Zhong et al also performed reconstructions with and without motion compensation. In a study including 6 patients at 3 T, they reported artifactually elevated apparent $R_{2}^{*}$ values in reconstructions without motion compensation (mean difference compared to Cartesian reference: 14.4 1/s), which was reduced by respiratory motion compensation (mean difference compared to Cartesian reference: 0.1 1/s). Moreover, they reported no substantial PDFF differences compared to the breath‐holding Cartesian reference technique.

In order to reduce reconstruction times, coil‐array compression was applied to compress the datasets into the first 8 (“Motion‐averaged") or 4 (“Motion‐resolved XD") eigenmodes. Heuristic observations showed that these compression factors considerably increase reconstruction speed without noticeably compromising image quality and quantification accuracy. However, future work should include determining the exact minimum number of eigenmodes needed for accurate PDFF and $R_{2}^{*}$ quantification for both proposed reconstructions.

## 6 CONCLUSION

Fat quantification from free‐breathing scans with radial k‐space sampling is feasible, both using motion‐averaged reconstruction and using motion‐resolved reconstruction. Moreover, simultaneous $R_{2}^{*}$ quantification is possible using motion‐resolved XD reconstruction. Therefore, the described free‐breathing technique might be a promising alternative to conventional Cartesian‐based methods for in vivo fat and iron quantification, which typically must be acquired during breath‐holds and which can be unreliable in patients who are unable to suspend respiration, such as sick, elderly, or pediatric patients. Further studies comprising a larger patient cohort with greater range of PDFF and $R_{2}^{*}$ values are needed to confirm these preliminary results.

## CONFLICT OF INTEREST

Hersh Chandarana receives hardware and software support from Siemens Healthcare GmbH. Manuel Schneider received PhD funding from Siemens Healthcare GmbH. Thomas Benkert, Dominik Nickel, Matthias Fenchel, and Berthold Kiefer are employees of Siemens Healthcare GmbH.

## Supporting information


**FIGURE S1** Effect of $\lambda_{W}$ and $\lambda_{F}$ regularization strength. In vivo patient maps of (A) the Cartesian reference scan and (B)‐(G) motion‐resolved free‐breathing maps for varying regularization weights $\lambda_{W}$, $\lambda_{F}$ and $\lambda_{R_{2}^{*}}\;=\;0$. Chosen $\lambda_{W}$ and $\lambda_{F}$ values are framed. The Dixon‐RAVE sequence did not apply “prescan normalize," resulting in a noticeable intensity drop in the water and fat maps toward the center compared to BH Cartesian (noticeable especially in the top row)
**FIGURE S2** Effect of $\lambda_{R_{2}^{*}}$ regularization strength on motion‐resolved maps. In vivo patient maps from (A) Cartesian reference scan and (B)‐(G) motion‐resolved reconstruction for varying $R_{2}^{*}$ regularization weights ( $\lambda_{W}\;=\;\lambda_{F}\;=\;0.4$). Maps of the chosen $\lambda_{R_{2}^{*}}$ value are framed. The Dixon‐RAVE sequence did not apply “prescan normalize,” resulting in a noticeable intensity drop in the water and fat maps toward the center compared to BH Cartesian (noticable especially in the top row)
**FIGURE S3** Example water, fat, $R_{2}^{*}$, and PDFF maps (from left to right) of the methods “Motion‐resolved XD" (top) and “Motion‐averaged" (bottom) from a patient (male, age: 25 years, weight: 78 kg, BMI: $24.6\;\text{kg}\;m^{-2}$). This animation is additionally included in a separate file (SupplFig3.gif)
**FIGURE S4** Example water, fat, $R_{2}^{*}$, and PDFF maps (from left to right) of the methods “Motion‐resolved XD" (top) and “Motion‐averaged" (bottom) for a patient (female, age: 50 years, weight: 59.9 kg, BMI: $24.1\;\text{kg}\;m^{-2}$) with elevated PDFF and $R_{2}^{*}$. This animation is additionally included in a separate file (SupplFig4.gif)
**FIGURE S5** Estimated respiratory signal (A), and PDFF (top) and $R_{2}^{*}$ (bottom) maps of a patient with slightly elevated $R_{2}^{*}$ values, reconstructed using the motion‐averaged reconstruction (B). Motion‐resolved XD reconstruction for frame 1 (end‐expiration) to frame 4 (end‐inspiration) are shown in (C)‐(F). The motion‐resolved XD parameter maps corresponding to frame 2 are selected for quantitative evaluation
**FIGURE S6** Bland‐Altman plots for the measured (left) PDFF and (right) $R_{2}^{*}$ values, depicting the agreement of (A) motion‐averaged reconstructions to the Cartesian reference, (B) motion‐gated (25% acceptance rate) reconstructions followed by NUFFT and image‐based water‐fat separation to the Cartesian reference, and (C) motion‐resolved XD reconstructions to the Cartesian reference. The plots indicate overall biases, 95% LoA and their 95% confidence intervals
