# Supplementary material for: Free‐breathing fat and R2* quantification in the liver using a stack‐of‐stars multi‐echo acquisition with respiratory‐resolved model‐based reconstruction
Source: Magn Reson Med. 2020 Apr 17;84(5):2592–605. doi: 10.1002/mrm.28280 (PMC7396291; doi:10.1002/mrm.28280)
Supplement: Supplementary file 1 — FIGURE S1 Effect of λW and λF regularization strength. In vivo patient maps of (A) the Cartesian reference scan and (B)‐(G) motion‐resolved free‐breathing maps for varying regularization weights λW, λF and λR2∗=0. Chosen λW and λF values are framed. The Dixon‐RAVE sequence did not apply “prescan normalize," resulting in a noticeable intensity drop in the water and fat maps toward the center compared to BH Cartesian (noticeable especially in the top row) FIGURE S2 Effect of λR2∗ regularization strength on motion‐resolved maps. In vivo patient maps from (A) Cartesian reference scan and (B)‐(G) motion‐resolved reconstruction for varying R2∗ regularization weights ( λW=λF=0.4). Maps of the chosen λR2∗ value are framed. The Dixon‐RAVE sequence did not apply “prescan normalize,” resulting in a noticeable intensity drop in the water and fat maps toward the center compared to BH Cartesian (noticable especially in the top row) FIGURE S3 Example water, fat, R2∗, and PDFF maps (from left to right) of the methods “Motion‐resolved XD" (top) and “Motion‐averaged" (bottom) from a patient (male, age: 25 years, weight: 78 kg, BMI: 24.6kgm-2). This animation is additionally included in a separate file (SupplFig3.gif) FIGURE S4 Example water, fat, R2∗, and PDFF maps (from left to right) of the methods “Motion‐resolved XD" (top) and “Motion‐averaged" (bottom) for a patient (female, age: 50 years, weight: 59.9 kg, BMI: 24.1kgm-2) with elevated PDFF and R2∗. This animation is additionally included in a separate file (SupplFig4.gif) FIGURE S5 Estimated respiratory signal (A), and PDFF (top) and R2∗ (bottom) maps of a patient with slightly elevated R2∗ values, reconstructed using the motion‐averaged reconstruction (B). Motion‐resolved XD reconstruction for frame 1 (end‐expiration) to frame 4 (end‐inspiration) are shown in (C)‐(F). The motion‐resolved XD parameter maps corresponding to frame 2 are selected for quantitative evaluation FIGURE S6 Bland‐Altman plots for the measured (left) PDFF [file MRM-84-2592-s001.pdf]

Supporting Information: Gradient of Non-Linear Cost Function

Minimizing Eq. (1) with a gradient-based technique requires calculating its gradient. An analytical formulation for $\nabla f = \left[\partial_{\boldsymbol{W}} f, \partial_{\boldsymbol{F}} f, \partial_{\boldsymbol{R}_2^*} f\right]$ of the data consistency term

$$f = \sum_{c,t_n} \| E(\boldsymbol{W}, \boldsymbol{F}, \boldsymbol{R}_2^*)_{c,t_n} - \boldsymbol{Y}_{c,t_n} \|_2^2 = \sum_{c,t_n} \| \boldsymbol{R}_{c,t_n} \|_2^2$$

can be derived following the description in (1). For simplification, $\boldsymbol{R}_{c,t_n}$ is used here to denote the data residual. The gradient with respect to water, fat, and R_2^* can then be calculated with

$$\frac{\partial f}{\partial \mathbf{W}} = \sum_{c,t_n} \mathrm{e}^{-\boldsymbol{R}_2^* t_n} \operatorname{Re}\left\{ \overline{\boldsymbol{C}_c}\, \mathrm{e}^{-2\pi i \boldsymbol{\Phi} t_n}\, \mathrm{FT}^{-1}\left(\boldsymbol{R}_{c,t_n}\right)\right\},$$

$$\frac{\partial f}{\partial \mathbf{F}} = \sum_{c,t_n} \mathrm{e}^{-\boldsymbol{R}_2^* t_n} \operatorname{Re}\left\{ \overline{\boldsymbol{C}_c}\, \mathrm{e}^{-2\pi i \boldsymbol{\Phi} t_n}\, \mathrm{FT}^{-1}\left(\overline{\boldsymbol{D}_{t_n}} \boldsymbol{R}_{c,t_n}\right)\right\},$$

and

$$\begin{aligned}\frac{\partial f}{\partial \boldsymbol{R}_2^*} = &\sum_{c,t_n} (-t_n)\, \mathrm{e}^{-\boldsymbol{R}_2^* t_n} \operatorname{Re}\left\{ \overline{\boldsymbol{C}_c}\, \mathrm{e}^{-2\pi i \boldsymbol{\Phi} t_n}\, \overline{\boldsymbol{W}}\, \mathrm{FT}^{-1}\left(\boldsymbol{R}_{c,t_n}\right)\right\} \\ &+ \sum_{c,t_n} (-t_n)\, \mathrm{e}^{-\boldsymbol{R}_2^* t_n} \operatorname{Re}\left\{ \overline{\boldsymbol{C}_c}\, \mathrm{e}^{-2\pi i \boldsymbol{\Phi} t_n}\, \overline{\boldsymbol{F}}\, \mathrm{FT}^{-1}\left(\overline{\boldsymbol{D}_{t_n}} \boldsymbol{R}_{c,t_n}\right)\right\}.\end{aligned}$$

Here, $\overline{A}$ corresponds the complex conjugate of A, and $\mathrm{FT}^{-1}(.)$ performs an inverse non-uniform fast Fourier transform (NUFFT). For minimizing the ℓ_1 norm of the regularization terms, the reader is referred to (2).

References

1. Block KT, Uecker M, Frahm J. Model-based iterative reconstruction for radial fast spin-echo MRI. IEEE Trans Med Imag 2009;28(11):1759–1769.

2. Lustig M, Donoho D, Pauly JM. Sparse MRI: The application of compressed sensing for rapid MR imaging. Magn Reson Med 2007;58(6):1182–1195.

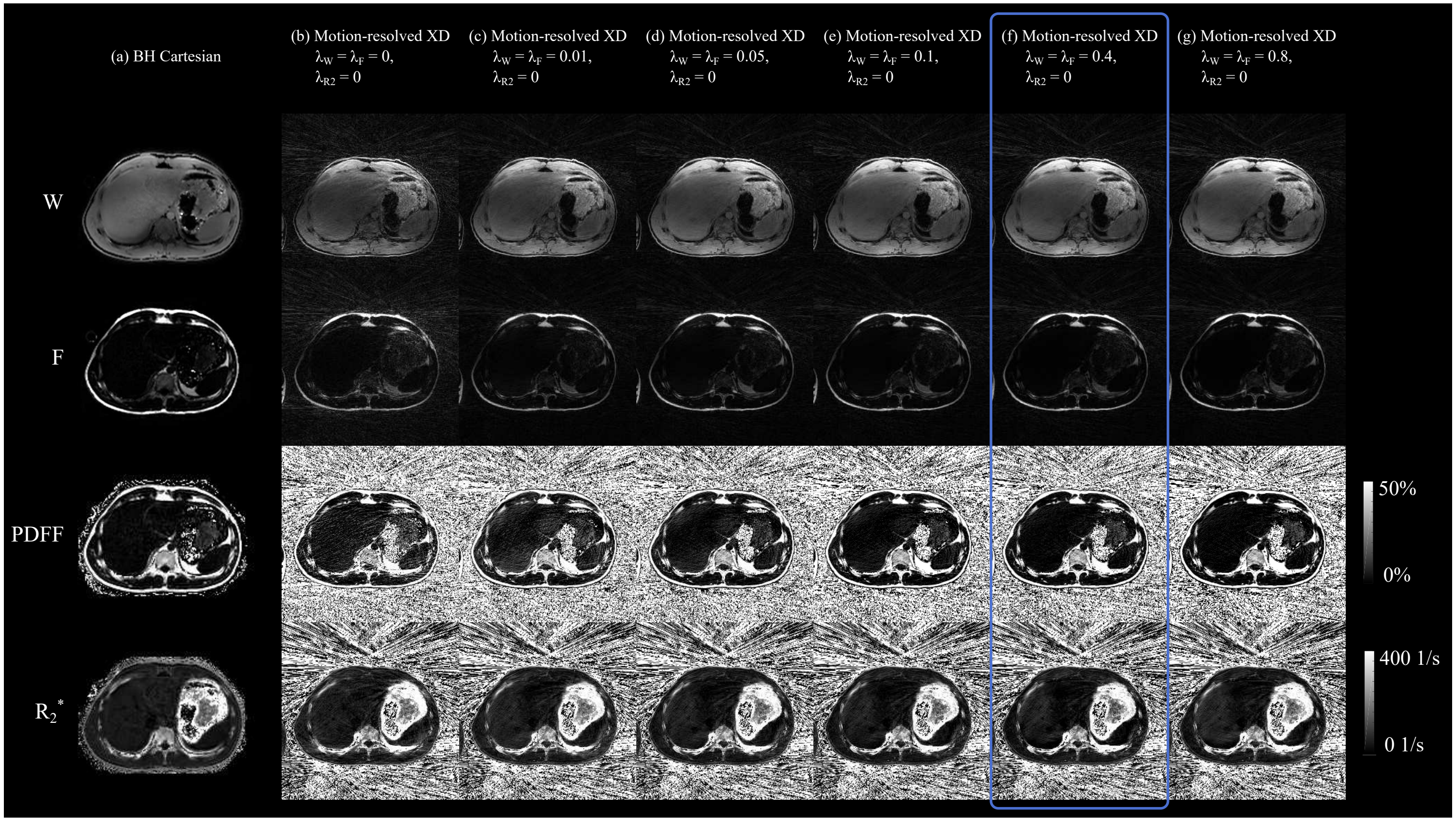

Supporting Information Figure S1. Effect of λ_W and λ_F regularization strength. In-vivo patient maps of (**a**) the Cartesian reference scan and (**b**)-(**g**) motion-resolved free-breathing maps for varying regularization weights λ_W, λ_F and $\lambda_{R_2^*} = 0$. Chosen λ_W and λ_F values are framed. The Dixon-RAVE sequence did not apply "prescan normalize", resulting in a noticeable intensity drop in the water and fat maps towards the center compared to BH Cartesian (noticable especially in the top row).

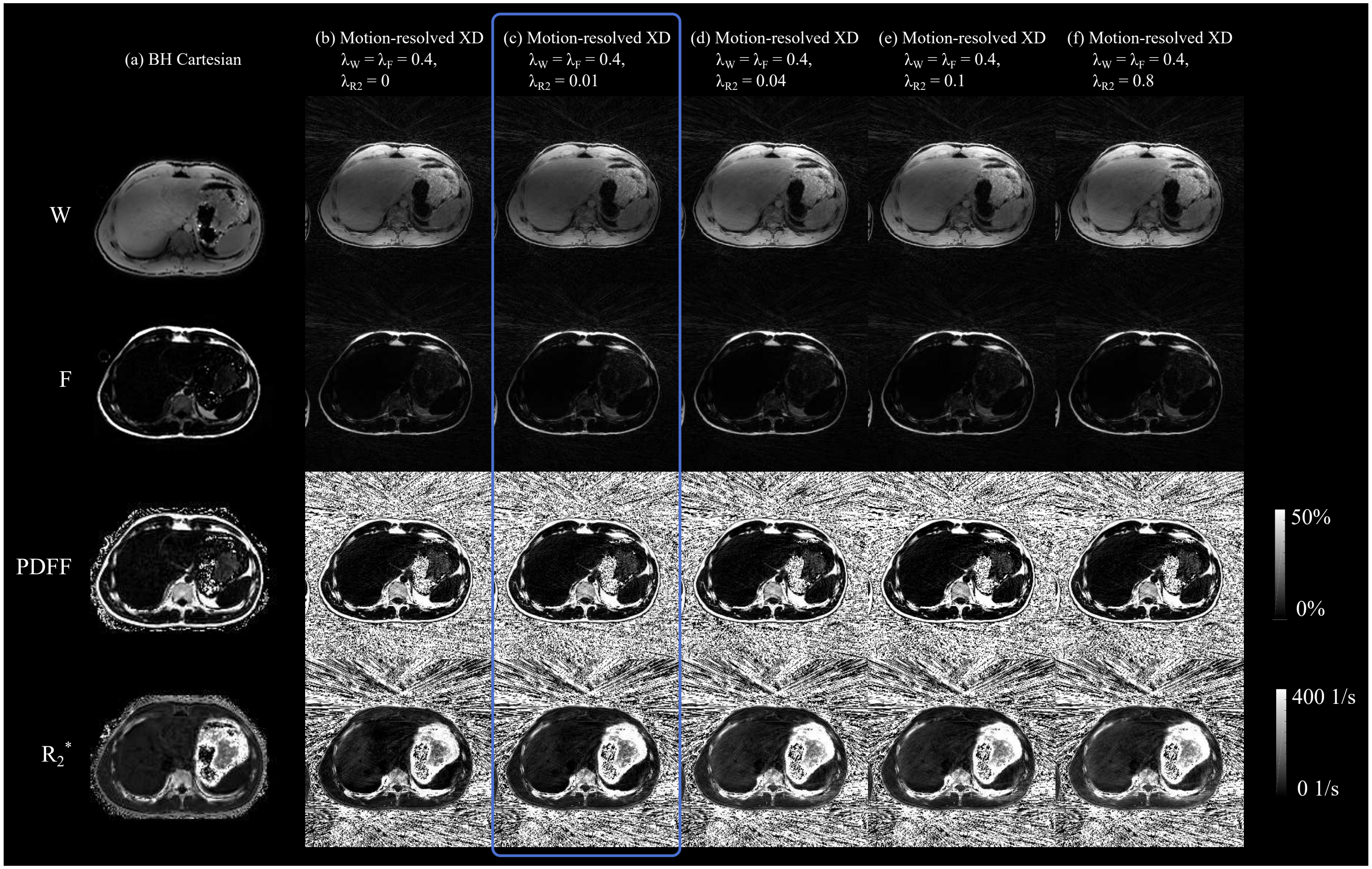

Supporting Information Figure S2. Effect of $\lambda_{R_2^*}$ regularization strength on motion-resolved maps. In-vivo patient maps from (**a**) Cartesian reference scan and (**b**)-(**g**) motion-resolved reconstruction for varying R_2^* regularization weights ($\lambda_W = \lambda_F = 0.4$). Maps of the chosen $\lambda_{R_2^*}$ value are framed. The Dixon-RAVE sequence did not apply "prescan normalize", resulting in a noticeable intensity drop in the water and fat maps towards the center compared to BH Cartesian (noticable especially in the top row).

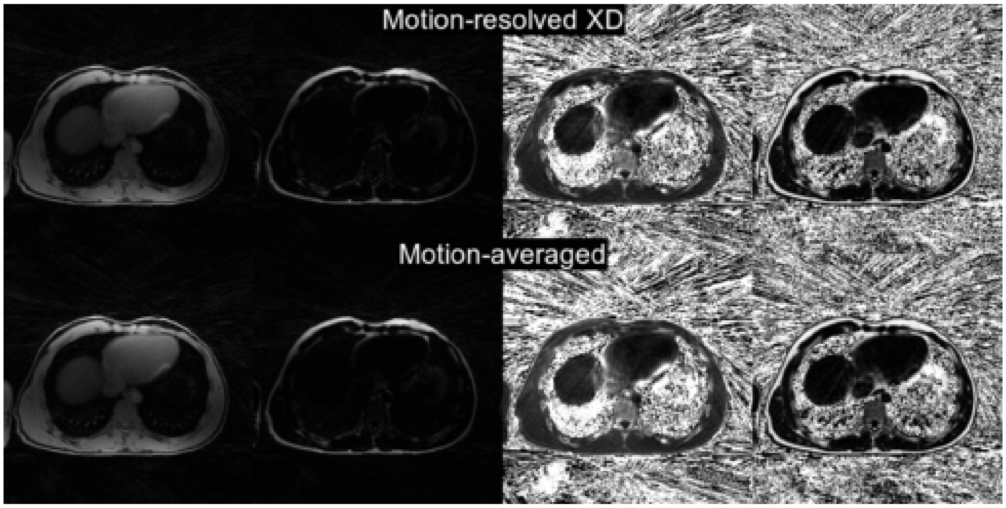

Supporting Information Figure S3. Example water, fat, R_2^*, and PDFF maps (from left to right) of the methods “Motion-resolved XD” (top) and “Motion-averaged” (bottom) from a patient (male, age: 25 years, weight: 78 kg, BMI: $24.6\,\mathrm{kg\,m^{-2}}$). This animation is additionally included in a separate file (SupplFig3.gif).

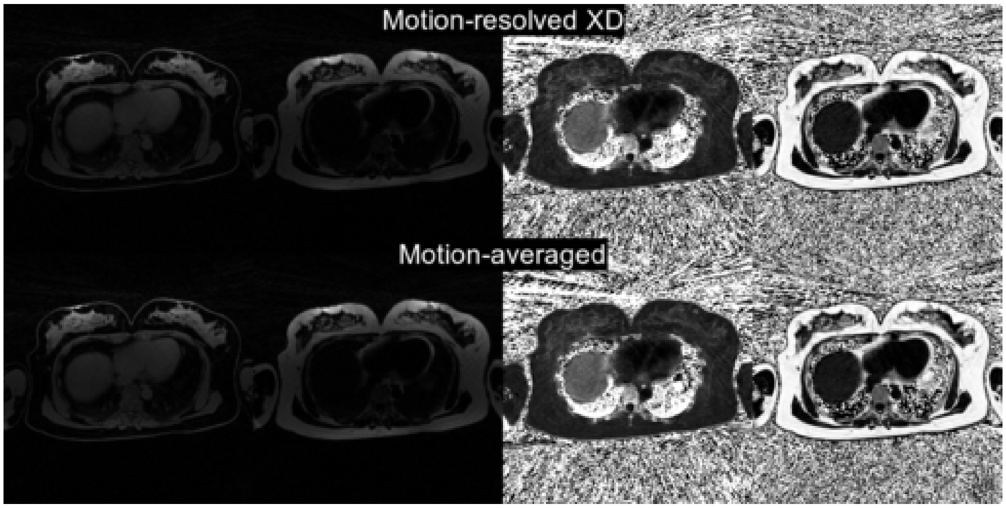

Supporting Information Figure S4. Example water, fat, R_2^*, and PDFF maps (from left to right) of the methods “Motion-resolved XD” (top) and “Motion-averaged” (bottom) for a patient (female, age: 50 years, weight: 59.9 kg, BMI: $24.1\,\mathrm{kg\,m^{-2}}$) with elevated PDFF and R_2^*. This animation is additionally included in a separate file (SupplFig4.gif).

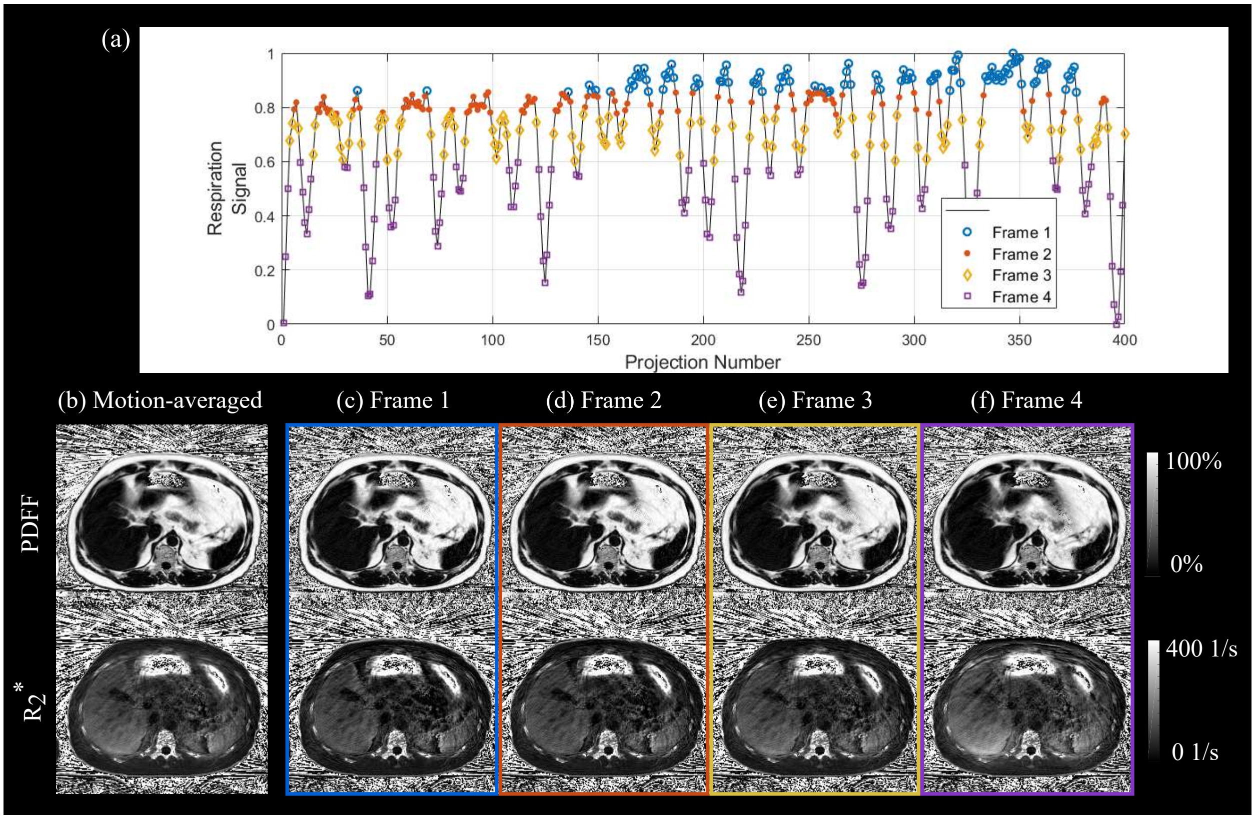

Supporting Information Figure S5. Estimated respiratory signal (**a**), and PDFF (top) and R_2^* (bottom) maps of a patient with slightly elevated R_2^* values, reconstructed using the motion-averaged reconstruction (**b**). Motion-resolved XD reconstruction for frame 1 (end-expiration) to frame 4 (end-inspiration) are shown in (**c**)-(**f**). The motion-resolved XD parameter maps corresponding to frame 2 are selected for quantitative evaluation.

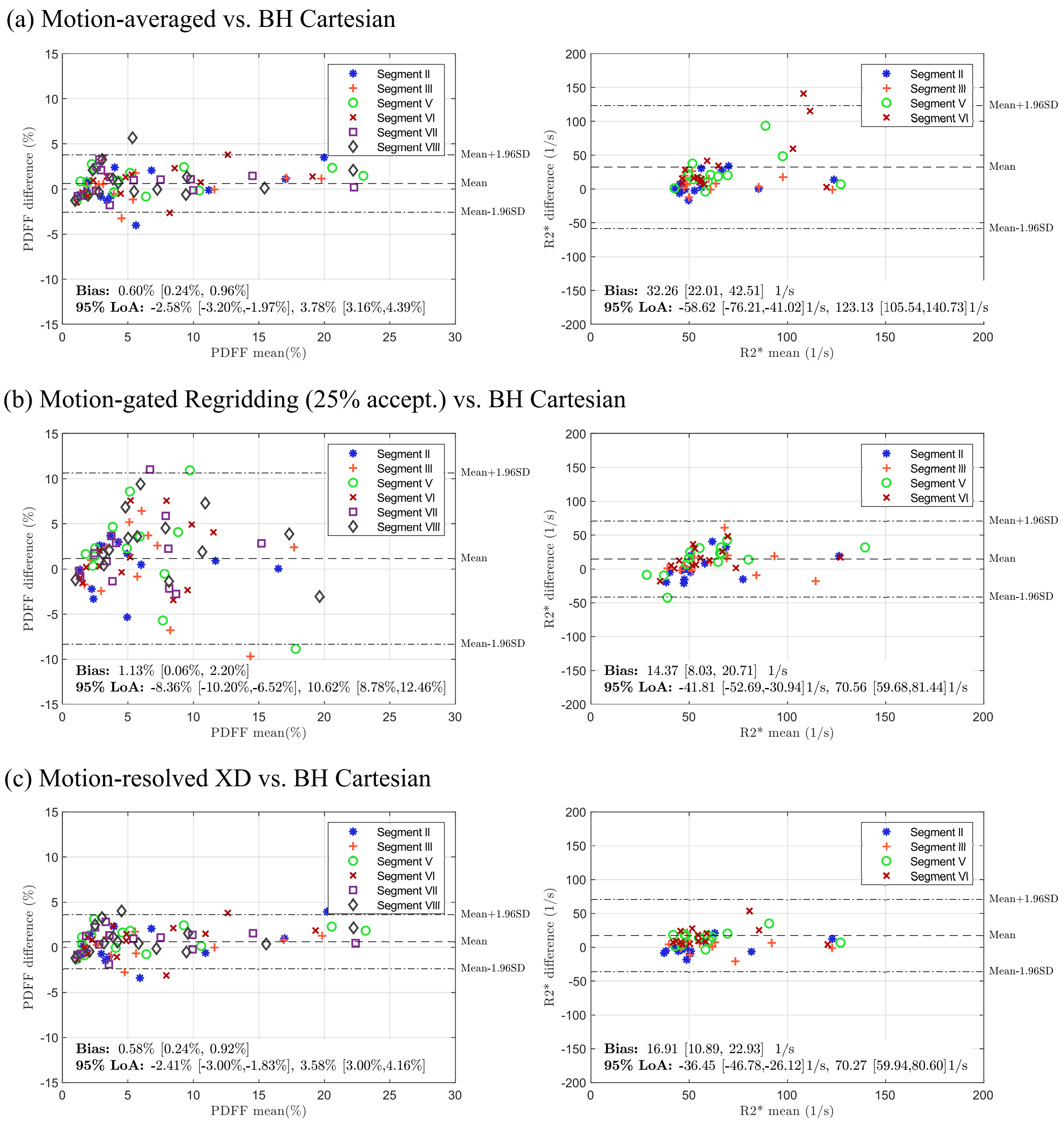

Supporting Information Figure S6. Bland-Altman plots for the measured (left) PDFF and (right) R_2^* values, depicting the agreement of (**a**) motion-averaged reconstructions to the Cartesian reference, (**b**) motion-gated ($25\,\%$ acceptance rate) reconstructions followed by regridding and image-based water/fat separation to the Cartesian reference, and (**c**) motion-resolved XD reconstructions to the Cartesian reference. The plots indicate overall biases, 95% LoA and their 95% confidence intervals.
